# MicroRNA-204 modulates colorectal cancer cell sensitivity in response to 5-fluorouracil-based treatment by targeting high mobility group protein A2

**DOI:** 10.1242/bio.015008

**Published:** 2016-04-19

**Authors:** Haijun Wu, Yu Liang, Lin Shen, Liangfang Shen

**Affiliations:** Department of Oncology, Xiangya Hospital, Central South University, 87 Xiang Ya Road, Changsha 410008, China

**Keywords:** miR-204, Colorectal cancer, HMGA2, 5-Fu

## Abstract

MicroRNAs (miRNAs) are a conserved class of ∼22 nucleotide RNAs that playing important roles in various biological processes including chemoresistance. Recently, many studies have revealed that miR-204 is significantly attenuated in colorectal cancer (CRC), suggesting that this miRNA may have a function in CRC. However, whether miR-204 modulates chemosensitivity to 5-fluorouracil (5-Fu) in colorectal cancer is still unclear. In our present study, we discuss this possibility and the potential mechanism exerting this effect. We identified high mobility group protein A2 (HMGA2) as a novel direct target of miR-204 and showed that miR-204 expression was decreased while HMGA2 expression was increased in CRC cell lines. Additionally, both MiR-204 overexpression and HMGA2 inhibition attenuated cell proliferation, whereas forced expression of HMGA2 partly restored the inhibitory effect of miR-204 on HCT116 and SW480 cells. Moreover, the miR-204/HMGA2 axis modulated the resistance of tumor cells to 5-Fu in HCT-116 and SW480 colon cancer cells via activation of the PI3K/AKT pathway. These results demonstrate that the miR-204/HMGA2 axis could play a vital role in the 5-Fu resistance of colon cancer cells. Taken together, our present study elucidated that miR-204 upregulated 5-Fu chemosensitivity via the downregulation of HMGA2 in colorectal cancer and provided significant insight into the mechanism of 5-Fu resistance in colorectal cancer patients. More importantly, our present study suggested that miR-204 has potential as a therapeutic strategy for 5-Fu-resistant colorectal cancer.

## Abstract (precis)

**Summary:** miR-204 upregulates 5-Fu chemosensitivity via the downregulation of HMGA2 in colorectal cancer and provides significant insight into the mechanism of 5-Fu resistance in colorectal cancer patients.

## INTRODUCTION

Nowadays, among diverse cancers, colorectal cancer is the third most common one and the third leading cause of cancer death in people worldwide (Siegel et al., 2015). Cancer inhibitors (tumor suppressors) and cancer inducers (oncogenes) are involved in the development of colorectal cancer. For the past 10 years, a class of noncoding RNA molecules known as microRNAs (miRNA) have been found to be associated with cancer development by acting as either tumor suppressors or oncogenes (Di Leva et al., 2014; Siegel et al., 2015). They are mainly endogenous, noncoding RNAs with a length of 19 to 24 nucleotides (Bartel, 2004). miRNAs deregulation has been involved in the evolution and progression of almost all kinds of tumors including colorectal cancer (Di Leva et al., 2014). According to many studies, miRNAs exert their effect on the gene expression downregulation via base pairing to complementary sites in the 3′-untranslated regions (UTR) of their target mRNAs (He and Hannon, 2004).

In colorectal cancer, many miRNAs expression patterns altered (Schetter et al., 2008), and some have been revealed to be associated with tumorigenesis by targeting tumor-associated genes (Huang et al., 2011; Liu et al., 2014; Wang et al., 2014). Moreover, miRNAs have been suggested to be promising tumor biomarkers (Ng et al., 2009; Huang et al., 2010). It was indicated that miR-204 most significantly negatively regulated in colorectal cancer tissues compared with adjacent noncancerous tissues. Previous studies have also reported that miR-204 was usually downregulated in many other cancers, which suggested a common role of miR-204 in human tumorigenesis (Gong et al., 2012; Mikhaylova et al., 2012; Bao et al., 2013; Yin et al., 2014). Yet the target gene of miR-204 in colorectal cancer remains undefined.

In present study, we identified high mobility group protein A2 (HMGA2) as a novel direct target of miR-204. Functional analyses showed that the miR-204/HMGA2 axis notably modulated cell proliferation and influenced the sensitivity of colorectal cancer cells to 5-fluorouracil (5-Fu). Further study on mechanism showed that the miR-204/HMGA2 axis played significant role on colorectal cancer development and progression via activating PI3K/AKT pathway. Taken together, data proposed the suppressive role of miR-204 on tumor and the consequences of its inactivation on the oncogenic activity of HMGA2. These factors could have marked contribution to the poor response of colorectal cancer to conventional chemotherapy.

## RESULTS

### HMGA2 mRNA is a direct target of miR-204

**Fig. 1.**
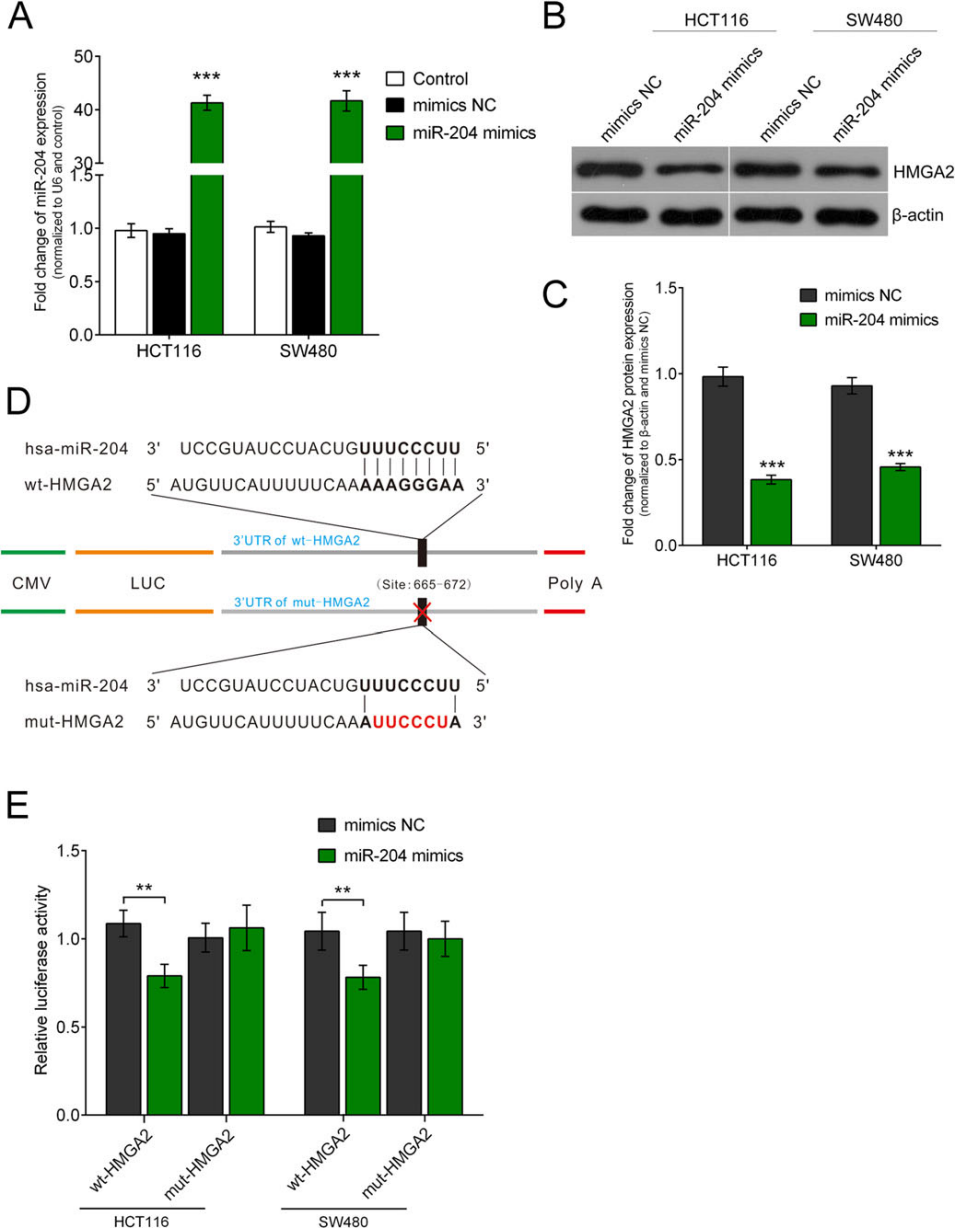
**Enhanced miR-204 CRC cell lines repressed HMGA2 protein expression and miR-204 directly targets HMGA2 by binding to its 3′ UTR.** (A-C) The results showed that, at 48 h after transfection, enhanced miR-204 in HCT116 and SW480 cell lines significantly repressed HMGA2 protein expression as compared with mimics NC. (D) We created a wt-HMGA2 3′ UTR luciferase reporter vector (wt-HMGA2), as well as a mut-HMGA2 3′ UTR luciferase reporter vector (mut-HMGA2) by sequentially mutating the predicted 8 bp miR-204 binding site in the HMGA2 3′ UTR. (E) The luciferase activity of the HMGA2 3′ UTR luciferase reporter vector was significantly reduced in miR-204 mimics transfected cells compared to scrambled control cells, while miR-204-mediated repression of HMGA2 3′ UTR luciferase reporter activity was abolished by mutation of the putative miR-204 binding site in the HMGA2 3′ UTR. Figure is representative of three experiments with similar results. Data represented as means±s.d.; **P<0.01, ***P<0.001 by one-way ANOVA.

We predicted potential targets of miR-204 using the TargetScan (http://www.targetscan.org/), miRanda (http://www.microrna.org/) and miRWalk (http://zmf.umm.uni-heidelberg.de/apps/zmf/mirwalk2/) online tools (Esquela-Kerscher and Slack, 2006; Hwang and Mendell, 2007; Manikandan et al., 2008) to clarify the molecular mechanism by which miR-204 exerts its inhibitory effect on HCT116 and SW480 cell line. Considering the involvement of HMGA2 in the pathogenesis of diverse human cancers, we identified HMGA2 as a key factor in this study among the predicted candidate targets. We transfected HCT116 and SW480 cell line with miR-204 mimics or mimics negative control (NC) to investigate whether miR-204 could inhibit HMGA2 expression. Results from western blot showed that, 48 h after transfection, increased miR-204 in HCT116 and SW480 cell lines notably suppressed HMGA2 protein expression as compared with mimics NC (Fig. 1A-C).

It was predicted by TargetScan, miRanda and miRWalk that miR-204 may use HMGA2 as a target by directly binding to its 3′ untranslated region. A wild-type HMGA2 3′ untranslated region luciferase reporter vector (wt-HMGA2), and a mutant-type HMGA2 3′ untranslated region luciferase reporter vector (mut-HMGA2) were created to confirm this prediction through a sequential mutation in the forecasted binding site of miR-204 in the *HMGA2* 3′ untranslated region (Fig. 1D). The wild-type HMGA2 vector and miR-204 mimics or inhibitor were co-transfected into HCT116 and SW480 cell line. Compared to scrambled control group, when co-transfected with miR-204 mimics, luciferase activity was significantly repressed (Fig. 1E). Conversely, miR-204-induced suppression of luciferase activity was eliminated when the two cell lines were co-transfected with miR-204 mimics and the mutant-type HMGA2 vector (Fig. 1E).

### The expression level of miR-204 is significantly inhibited in CRC

**Fig. 2.**
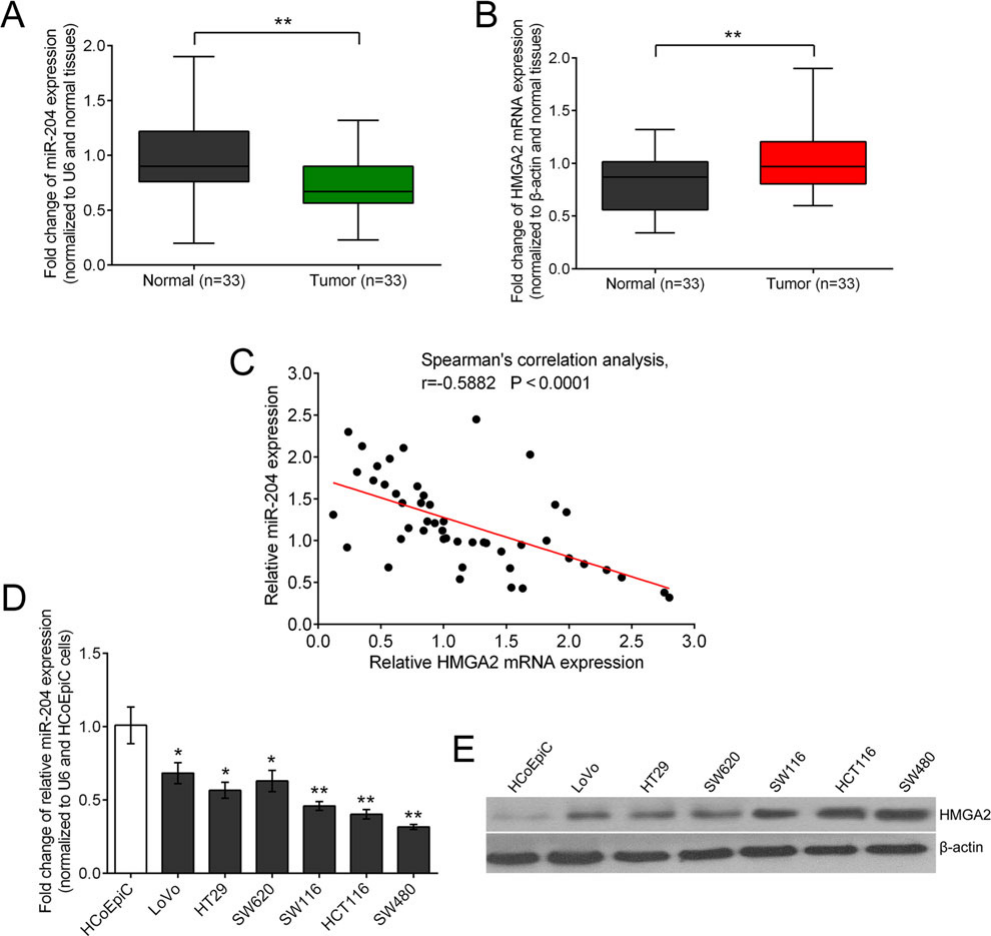
**MiR-204 is downregulated in both primary CRC tissues and CRC cell lines.** (A) The expression of miRNAs in the CRC tissues and the matched normal tissues was detected by qRT-PCR and normalized to that of U6. Results showed that the expression of miR-204 was significantly decreased in tumor tissue. (B) Expression of HMGA2 mRNA, normalized to β-actin, was increased compared with the matched normal tissue. (C) Spearman's correlation test showed a significant inverse correlation of the miR-204 and HMGA2 mRNA expression levels in CRC tissues. (D,E) miR-204 was expressed at significantly lower levels in six CRC cell lines while HMGA2 was expressed at higher levels in comparison with normal colonic mucosa cells. Figure is representative of three experiments with similar results. Data represented as means±s.d.; *P<0.05, **P<0.01 by one-way ANOVA.

Given that miR-204 could target *HMGA2* by directly binding to its 3′ untranslated region, further study was in progress to determine the expression levels of miR-204 and HMGA2 in both CRC and adjacent normal tissues (as the control group) by performing a qPCR assay. Results revealed that the expression levels of miR-204 were much lower in CRC tissues compared to that in the control group in 26/33 (78.79%) of samples, whereas HMGA2 expression was shown to be upregulated in 81.82% (27/33) of samples (Fig. 2A,B). We observed a reverse correlation between the mRNA expression levels of miR-204 and HMGA2 by performing Spearman's correlation test, r=−0.5882 (*P*<0.0001) (Fig. 2C), which confirmed that downregulation of miR-204 expression was clearly linked with upregulation of HMGA2 expression at the level of transcription in CRC tissues. Consistently, the expression level of miR-204 was notably repressed while the expression level of HMGA2 was increased in CRC cell lines compared with the normal cell line (Fig. 2D,E).

### miR-204 facilitates the inhibitory effect of 5-Fu on CRC cell lines

**Fig. 3.**
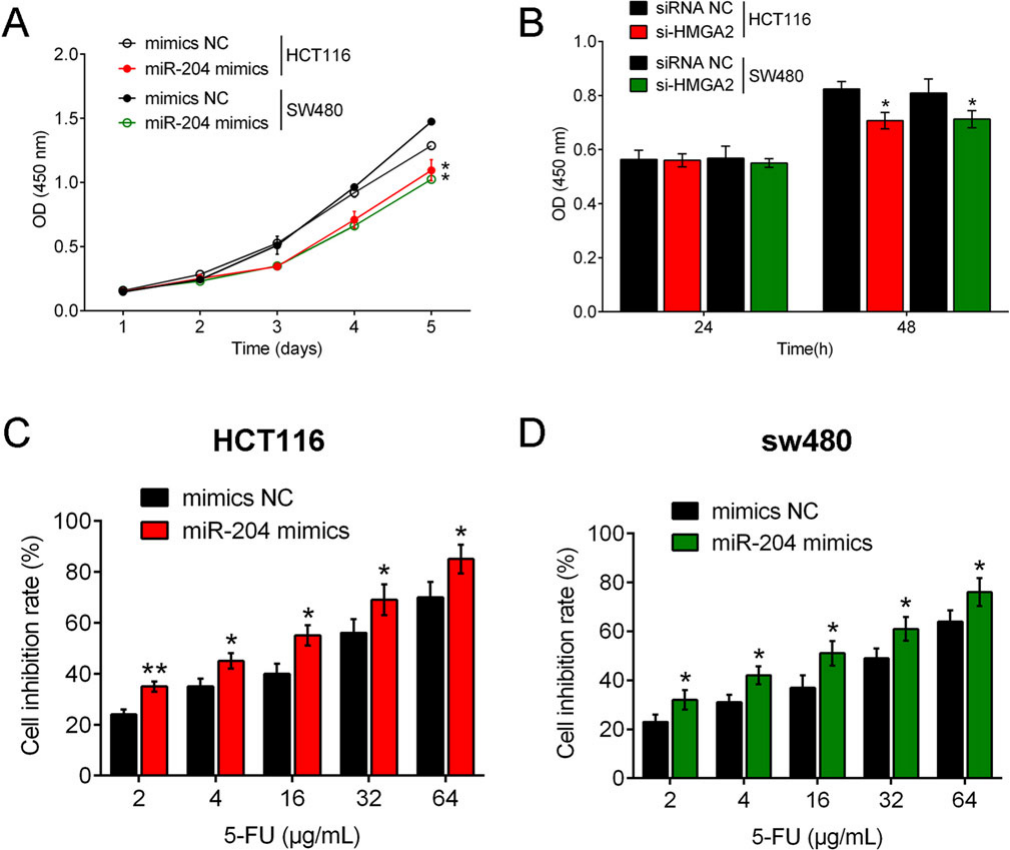
**miR-204 facilitates the inhibitory effect of 5-Fu on CRC cell lines.** (A) CRC cell proliferation was examined under conditions of miR-204 overexpression by miR-204 mimic transfection or HMGA2 inhibition by si-HMGA2 without 5-Fu treatment to verify the role of miR-204/HMGA2 in CRC cell growth. Results showed that proliferation of both HCT116 and SW480 cell lines were inhibited by miR-204 overexpression or HMGA2 inhibition compared with null-transfected cell lines. (B) Under a dose-dependent 5-Fu treatment at concentrations of 2, 4, 16, 32 and 64 μg/ml, proliferation levels of miR-204 mimic-transfected CRC cell lines were examined. Results showed that cell inhibition rate of HCT116 and SW480 cell lines increased with 5-Fu dose, and the cell inhibition rate of miR-204 mimic-transfected CRC cell lines was higher compared with null-transfected cell lines. Figure is representative of three experiments with similar results. Data represented as means±s.d.; *P<0.05, **P<0.01 by one-way ANOVA.

Previous studies have reported that miR-204 is usually downregulated in many cancers, and miR-204 expression was demonstrated to be reduced in six human CRC cell lines in the present study. CRC cell proliferation was examined under conditions of miR-204 overexpression by miR-204 mimic transfection or HMGA2 inhibition by si-HMGA2 without 5-Fu treatment to verify the role of miR-204/HMGA2 in CRC cell growth. Results showed that proliferation of both HCT116 and SW480 cell lines were inhibited by miR-204 overexpression or HMGA2 inhibition compared with null-transfected cell lines (Fig. 3A,B). Next, a dose-dependent 5-Fu treatment at concentrations of 2, 4, 16, 32 and 64 μg/ml was conducted on miR-204 mimic-transfected CRC cell lines and cell line growth was examined. Results showed that cell inhibition rate of HCT116 and SW480 cell lines increased with 5-Fu dose, and the cell inhibition rate of miR-204 mimic-transfected CRC cell lines was higher compared with null-transfected cell lines (Fig. 3C,D), suggesting 5-Fu treatment attenuated cell growth and miR-204 overexpression amplified the sensitivity of CRC cell lines to 5-Fu.

### Knockdown HMGA2 inhibits CRC cell growth in response to 5-Fu

**Fig. 4.**
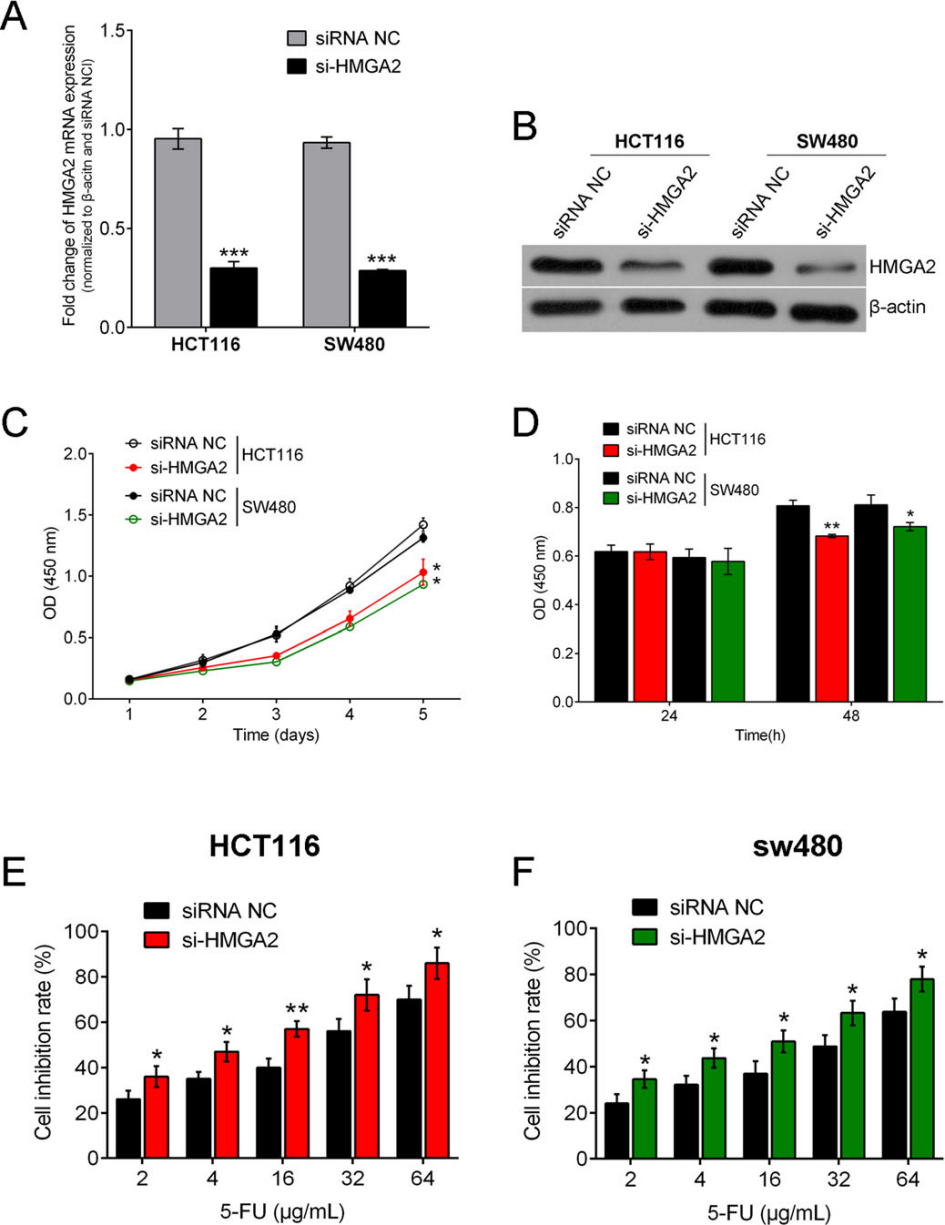
**Knockdown HMGA2 inhibits CRC cell growth in response to 5-Fu.** (A,B) HMGA2 inhibition was achieved by si-HMGA2 and the inhibitory efficiency was verified by real-time PCR and western blot. (C,D) MTT and BrdU assays were performed and results showed that the viabilities of HCT116 and SW480 cell lines were significantly abrogated when HMGA2 inhibited without 5-Fu treatment. (E,F) Under a dose-dependent 5-Fu treatment at concentrations of 2, 4, 16, 32 and 64 μg/ml, proliferation levels of HMGA2-inhibited CRC cell lines were examined. Cell inhibition rate of HCT116 and SW480 cell lines increased with 5-Fu dose, and the cell inhibition rate of HMGA2 inhibited CRC cell lines was higher compared with null- transfected cell lines. Data represented as means±s.d.; *P<0.05, **P<0.01, ***P<0.001 by one-way ANOVA.

To further confirm the miR-204/HMGA2 axis impacted in the regulation of CRC cell sensitivity to 5-Fu, we knock down HMGA2 and then examined the CRC cell viability to see whether knocking down HMGA2 can phenocopy the effects of miR-204. HMGA2 inhibition was achieved by si-HMGA2 and the inhibitory efficiency was verified by real-time PCR and western blot (Fig. 4A,B). MTT and BrdU assays were performed and results showed that the viabilities of HCT116 and SW480 cell lines were significantly abrogated when HMGA2 was inhibited without 5-Fu treatment (Fig. 4C,D). Then a dose-dependent 5-Fu treatment at concentrates of 2, 4, 16, 32 and 64 μg/ml was conducted on HMGA2-inhibited CRC cell lines and CRC cell line growth was examined. Similar results as in miR-204 overexpressed CRC cell lines were observed, that is, cell inhibition rates of HCT116 and SW480 cell lines increased with 5-Fu dose, and the cell inhibition rate of HMGA2 inhibited CRC cell lines was higher compared with null-transfected cell lines (Fig. 4E,F). Taken together, these data suggested that HMGA2 inhibition reduced CRC cell growth and exerted a similar effect to miR-204 overexpression.

### HMGA2 overexpression rescued the effects of miR-204 on CRC cells when exposed to 5-Fu treatment

**Fig. 5.**
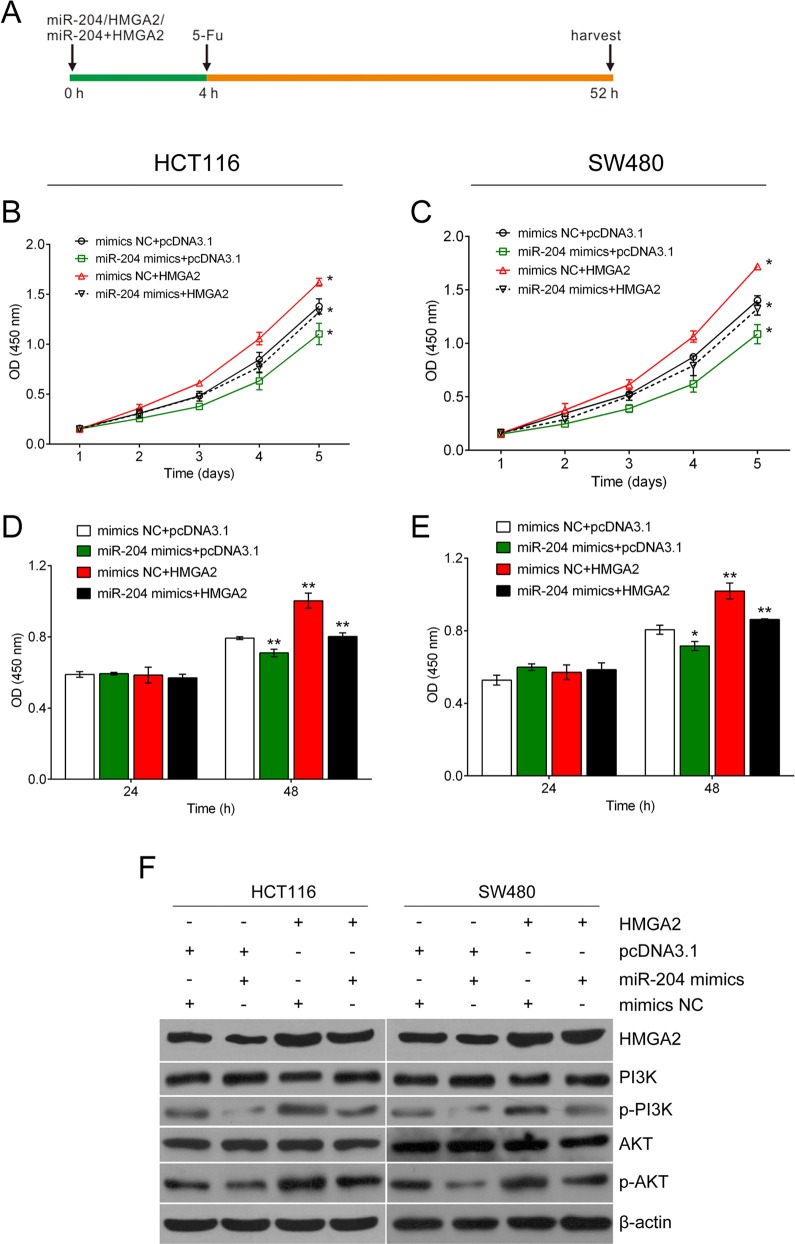
**Forced expression of HMGA2 restores the effects of miR-204 in CRC cells when exposed to 5-Fu treatment.** (A) Time axis of the experiment. The mimics NC, miR-204 mimics, pcDNA3.1 and HMGA2 ORF clone were given 4 h before 5-Fu (32 μg/ml) treatment. At 48 h after 5-Fu treatment, the cells were harvested for further analysis. (B-E) MTT and BrdU assays were used to determine cell proliferation. (F) PI3K, p-PI3K, AKT and p-AKT protein level were determined by western blot assay. Cell viability and PI3K/AKT pathway activation were significantly decreased in cells treated with miR-204 mimics compared to mimics NC group. Forced expression of HMGA2 restored significant inhibition of cell viability and PI3K/AKT pathway activation by miR-204 in HCT116 and SW480 cell lines when exposed to 5-Fu treatment. Figure is representative of three experiments with similar results. Data represented as means±s.d.; *P<0.05, **P<0.01 by one-way ANOVA.

Since the miR-204/HMGA2 axis could regulate the proliferation of CRC cell lines, we wondered whether it could further modulate the cell viability of HCT116 and SW480 cell lines under 5-Fu treatment. 5-Fu treatment at a concentration of 32 μg/ml was conducted 4 h after cell transfection (Fig. 5A). As shown in Fig. 4B-E, cell viability was significantly decreased in cells treated with miR-204 mimics compared to mimics NC group. Forced expression of HMGA2 restored, at least in part, the significant inhibitory effect of miR-204 on cell viability in HCT116 and SW480 cell lines exposed to 5-Fu treatment.

The PI3K/Akt signaling pathway mediates resistance to 5-Fu in human malignant tumors, including colon cancer (Shin et al., 2010; Chen et al., 2015; Das et al., 2015). Fig. 5C shows that overexpression of miR-204 inhibited the protein expression of p-PI3K and p-AKT but the total protein levels of PI3K and AKT stayed unchanged. Forced expression of HMGA2 restored the effect of miR-204 on HCT116 and SW480 cells under 5-Fu treatment. The results revealed that miR-204 could suppress the activation of the PI3K/AKT signaling pathway through HMGA2 in 5-Fu treated cells.

## DISCUSSION

HMGA2 plays a vital role in stimulating normal cardiogenesis (Monzen et al., 2008), and mouse central and peripheral neural stem cell self-renewal (Nishino et al., 2008), thereby regulating cell growth and differentiation. HMGA2 expression is elevated in many malignant neoplasms (Fusco and Fedele, 2007; Cleynen and Van de Ven, 2008), and its overexpression is a poor prognostic factor for colon cancer, lung cancer (Sarhadi et al., 2006), oral squamous cell carcinoma (Miyazawa et al., 2004), ovarian cancer (Kim et al., 2015), and metastatic breast cancer (Sun et al., 2015). HMGA2 expression has been reported to be involved in Dukes stages and metastasis of CRCs in a cross-section study (Li et al., 2015). Thus, targeted inhibition of HMGA2 may be crucial for the treatment of colon cancer.

Over the past decade, more and more studies have reported that miRNAs could regulate up to 60% of human protein-coding genes as important gene regulators (Friedman et al., 2009). According to the latest version of miRBase (Release 20: June 2013), 2578 human mature miRNAs have been annotated. Here, we focus on searching for a miRNA which can modulate HMGA2 expression, and then reveal its impacts on CRC cell growth. It has been predicted that miR-204 could target *HMGA2* by binding to its 3′ untranslated region. To verify this prediction, a luciferase reporter assay was performed. Results showed that co-transfection of miR-204 mimics and wild-type HMGA2 vector lead to an obvious reduction of luciferase activity, while co-transfection of miR-204 mimics and mutant-type HMGA2 vector eliminated the repression of luciferase activity, suggesting that miR-204 targets HMGA2 by directly binding to its 3′ untranslated region. Forced overexpression of miR-204 inhibited the expression of HMGA2 at both the mRNA and protein levels, and the inhibitory efficiency was enhanced as the precursor miRNA concentration increased. In addition, an inverse correlation between the expression levels of miR-204 and HMGA2 was observed both in the CRC tissues and cell lines. We showed that miR-204 reduced the endogenous HMGA2 protein content by regulating the HMGA2 expression at the level of transcription. Taken together, our results revealed that miR-204 plays a key role in regulating HMGA2 expression.

Previous studies have identified that MiR-204 plays a suppressive role in diverse malignant tumors through many signaling pathways (Gong et al., 2012; Bao et al., 2013; Ying et al., 2013), suggesting it may exert different effects in cancer initiation. For example, in the context of renal clear cell carcinoma (Mikhaylova et al., 2012) and pancreatic cancer (Chen et al., 2013), miR-204 could stop a tumor from growing. miR-204 also represses cancer cell invasion in endometrial cancer (Chung et al., 2012), glioma (Ying et al., 2013), colon cancer (Qiu et al., 2013), intrahepatic cholangiocarcinoma (Qiu et al., 2013), and head and neck tumors (Lee et al., 2010). In addition, miR-204 also plays important role in the sensitivity of cancer cell to chemotherapy drugs in neuroblastoma and gastric cancer through targeting BCL2 (Sacconi et al., 2012). Here, we revealed that miR-204 inhibited proliferation in colorectal cancer by directly targeting HMGA2. Proliferation levels of both HCT116 and SW480 cell lines were inhibited by miR-204 overexpression or HMGA2 inhibition compared with null-transfected cell lines. Furthermore, under dose-dependent 5-Fu treatment, cell inhibition rate of HCT116 and SW480 cell lines increased with 5-Fu dose in miR-204 overexpressed CRC cells, and the cell inhibition rates of both miR-204 overexpressed CRC cell lines were higher compared with null-transfected cell lines, suggesting 5-Fu treatment attenuated cell growth and miR-204 overexpression amplified the sensitivity of CRC cell lines to 5-Fu.

To further confirm the role of the miR-204/HMGA2 axis in the regulation of CRC cell sensitivity to 5-Fu, we knocked down HMGA2 and then examined CRC cell viability to see whether knocking down HMGA2 can phenocopy the effects of miR-204. As expected, the viabilities of HCT116 and SW480 cell lines were both significantly abrogated when HMGA2 inhibited. Consequently, under dose-dependent 5-Fu treatment, similar results were observed in HMGA2-inhibited CRC cell lines; in that cell inhibition rate of HCT116 and SW480 cell lines increased with 5-Fu dose, and the cell inhibition rate of HMGA2-inhibited CRC cell lines was higher compared with null-transfected cell lines. Taken together, these results suggest that miR-204/HMGA2 plays key role in regulating chemoresistance of CRC cell to 5-Fu.

Despite of the successful treatment of colorectal cancer, resistance to conventional chemotherapy still remains a critical obstacle. Most chemotherapeutic agents suppress cell viability to eventually kill tumor cells, including oxaliplatin, 5-Fu, DDP, and adriamycin, via distinct antitumor mechanisms (Kerr et al., 1994; Dalianis, 2014). In the present study, we showed miR-204 could promote the sensitivity of colorectal cancer cells to the most commonly used drug (5-Fu) for colorectal cancer by targeting HMGA2, which suggested significant potential for miR-204/HMGA2 signaling in cell viability regulation. This pathway could be a promising new target for preventive and therapeutic strategies of colorectal cancer.

A variety of mechanisms, including intrinsic cellular resistance and microenvironmental interactions, have been reported to explain the involvement of PI3K/AKT pathway-mediated resistance to 5-Fu in human malignant tumors (Shin et al., 2010; Das et al., 2015). Activated PI3K/AKT pathway increases the survival of tumor cells *in vitro*, which identify PI3K/AKT pathway as a key role in 5-Fu resistance (Allen et al., 2013; Chen et al., 2015). In this study, western blotting showed that with 5-Fu treatment, the expression of p-PI3K and p-Akt protein were reduced in the miR-204-transfected group, and induced in the HMGA2-transfected group. Thus, the miR-204/HMGA2 axis regulated the activity of the PI3K/Akt signaling pathway in the 5-Fu-treated CRC cells.

In conclusion, we have identified an important tumor-suppressive miRNA, miR-204, that is frequently downregulated in human colorectal cancer. MiR-204 plays key roles in colorectal cancer development and progression, by repressing colorectal cancer cell growth as well as by promoting colorectal cancer cell sensitivity to a chemotherapeutic drug (5-Fu) through directly targeting HMGA2. We also demonstrated that the miR-204/HMGA2 axis regulates colorectal cancer cell sensitivity to 5-Fu partly through the PI3K/AKT signaling pathway. Therefore, our study demonstrates the importance of miR-204/HMGA2 signaling in colorectal cancer tumorigenesis and suggests that targeting this signaling may represent a new therapeutic approach for human colorectal cancer.

## MATERIALS AND METHODS

### Tissue samples, cell lines and cell transfection

We collected a total of 33 paired primary CRC tissues and the matched adjacent normal colonic epithelial tissues from patients who underwent surgical resection at Xiangya Hospital of Central South University (Changsha, China). This project was approved by the Ethics Committee of Xiangya Hospital of Central South University. Informed consent in the study was obtained from the participants. We snap froze all the samples in liquid nitrogen, and then stored them at −80°C.

We purchased Human Colonic Epithelial Cell (HcoEpiC) and six human CRC cell lines, LoVo, HT29, SW620, SW116, HCT116 and SW480 cells from the American Type Culture Collection (Manassas, VA, USA) and cultured them in RPMI-1640 medium (Invitrogen, CA, USA) supplemented with 10% fetal bovine serum (Gibco, CA, USA) at 37°C in a humidified atmosphere with 5% CO_2_. By transfection of miR-204 lentivirus we achieved ectopic overexpression of miR-204 (Genepharma, Shanghai, China) using Lipofectamine 2000 (Invitrogen). By using an HMGA2 ORF-expressing clone we achieved overexpression of HMGA2 (GeneCopoecia, Guangzhou, China). HMGA2 inhibition was achieved by si-HMGA2. We plated cells in 6-well plates or 96-well plates, transfected and incubated them for 24 h or 48 h and used the cells for further assays or RNA/protein extraction.

### RNA extraction and quantitative real-time RT-PCR

Total RNA was extracted from 10-20 mg of tumor samples and from 30-40 mg of normal tissues. Samples were mechanically disrupted and simultaneously homogenized in the presence of QIAzol Lysis reagent (Qiagen, CA, USA), using a Mikrodismembrator (Braun Biotech International, Melsungen, Germany). RNA was extracted using the miRNeasy Mini kit (Qiagen) according to manufacturer's instructions.

Approximately 1.0×10^6^ SW480 or HCT116 cells (uninfected or infected) were seeded into 6-well culture plates, cultured for 48 h and harvested. Small RNAs (∼200 nt) were isolated using the mirVanaTM PARIS TM Kit (Ambion, CA, USA) according to the manufacturer's instructions.

For reverse transcription (RT) reactions, 1 mg of small RNAs was reverse transcribed with the miScript Reverse Transcription Kit (Qiagen) at 37°C for 60 min followed by a final incubation at 95°C for 5 min. miRNA real-time RT-PCR was carried out using the miScript SYBR Green PCR kit (Qiagen) on an CFX96 real-time PCR machine (Bio-Rad, CA, USA). PCR was conducted at 95°C for 15 min, followed by 40 cycles of 94°C for 15 s, 55°C for 30 s and 70°C for 30 s. The expression of each miRNA was normalized to U6 snRNA.

Expression of HMGA2 mRNA was detected by quantitative real-time RT-PCR (qRT-PCR) using the standard SYBR Green RT-PCR Kit (Bio-Rad) according to the manufacturer's instructions. Briefly, total RNA was extracted from the cells using TRIzol reagent (Invitrogen) and cDNA was synthesized using the RevertAid First-Strand cDNA Synthesis kit (Fermentas, CA, USA), according to the manufacturer's protocol. Each cDNA sample was used as a template for PCR in triplicate with iQTM SYBR Green Supermix (Bio-Rad) by denaturation at 94°C for 1 min; 30 cycles of 94°C for 40 and 60°C for 40 s; followed by extension at 72°C for 6 min. The specific primer pairs were: HMGA2 (107 bp), sense: 5′-GGC GGT GAA GGA GAT GAA C-3′; antisense: 5′-TGA TGA GGA AAT CCA CGA TAG AG-3′; β-actin (202 bp) sense: 5′-GGC GGC ACC ACC ATG TAC CCT-3′; reverse: 5′-AGG GGC CGG ACT CGT CAT ACT-3′. The relative levels of HMGA2 mRNA were normalized to the internal control β-actin. Relative gene expression was quantified using CFX Manager software (Bio-Rad) and expressed as percentage of control cells.

### Western blotting

We lysed cells cultured in 35 mm dishes in 0.2 ml lysis buffer [0.1% SDS, 1% NP-40, 50 mM HEPES, pH 7.4, 2 mM EDTA, 100 mM NaCl, 5 mM sodium orthovanadate, 40 μM p-nitrophenyl phosphate and 1% protease inhibitor mixture set I (Calbiochem, USA)]. After centrifuging lysates at 10000 ***g*** for 15 min, we collected the supernatants, denatured, separated them using 10% SDS-PAGE gels and blotted them onto polyvinylidene difluoride membranes. We blocked the membranes in 5% albumin from bovine serum (BSA) for 1.5 h at room temperature, then probed them with 1:1000 diluted rabbit polyclonal HMGA2 (Abcam, MA, USA) at 4°C overnight, then subsequently incubated the blots with HRP-conjugated secondary antibody (1:5000). ECL Substrates were used to visualize signals (Millipore, MA, USA). β-actin was used as an endogenous protein for normalization.

### MTT assay

A modified MTT assay was used to evaluate cell viability. We assessed the viability of SW480 or HCT116 cells transfected with miR-204 or control at five time points (on day 1, 2, 3, 4 and 5) after 2×10^3^ transfected cells/well seeded into 96-well culture plates. Mainly, we achieved quantification of mitochondrial dehydrogenase activity via the enzymatic conversion of MTT [3-(4,5-dimethyldiazol-2-yl)-2,5- diphenyltetrazolium bromide; Sigma-Aldrich, MO, USA] to a colored formazan product. We added MTT (10 μl, 10 mg/ml) to the cells, incubated the mix for 4 h, and terminated the reaction by removing the supernatant and adding 100 μl DMSO to dissolve the formazan product. After 0.5 h, we used a plate reader at 490 nm to measure the optical density (OD) of each well (ELx808 Bio-Tek Instruments, USA).

### BrdU incorporation assay

We performed BrdU assays at 24 h and 48 h after transfecting SW480 or HCT116 cells with miR-204 or control vector to determine DNA synthesis in proliferating cells. We seeded infected cells in 96-well culture plates at a density of 2×10^3^ cells/well, cultured them for 24 h or 48 h, and incubated them with a final concentration of 10 μM BrdU (BD Pharmingen, CA, USA) for 2 h to 24 h. At the end of the incubation period, we removed the medium and fixed the cells for 30 min at RT, incubated them with peroxidase-coupled anti-BrdU-antibody (Sigma-Aldrich) for 60 min at RT, washed them for three times with PBS, incubated them with peroxidase substrate (tetramethylbenzidine) for 30 min, and finally measured the absorbance values at 490 nm. Background BrdU immunofluorescence was determined in cells not exposed to BrdU but stained with the BrdU antibody.

### 3′ UTR luciferase reporter assay

We performed UTR luciferase reporter assays in human CRC cells. Vectors based on pMIR-REPORT harboring the wild-type (WT) 350 bp fragment of the *HMGA2* 3′ UTR, or the same fragment in which the miR-204 binding site (199-194) was mutated (MUT), were inserted downstream of the luciferase reporter gene stop codon in pMIR-REPORT using *Hind*III and *Spe*I. The cells were co-transfected with (1) miR-204 mimics or mimics NC (50 nM), (2) pMIR-REPORT vectors containing the WT or MUT miR-204 binding sites (400 ng) and (3) pRL-SV40 (Promega, CA, USA) expressing *Renilla* luciferase (400 ng) for normalization of transfection efficiency. We grew cells in high-glucose DMEM supplemented with 10% fetal bovine serum, and measured luciferase activities at 48 h post-transfection using the Dual-Luciferase Reporter Assay System (Promega).

### Statistical analysis

We expressed data as mean±s.d. of three independent experiments and processed data by using SPSS 17.0 statistical software (SPSS, IL, USA). By using Wilcoxon's paired test we compared the expression of miR-204 in CRC tissues and the paired adjacent normal colonic tissues. One-way ANOVA was used to evaluate the differences between groups in the migration and invasion assays. *P* values of <0.05 were considered statistically significant.
